# Objective Quantification of Neuromotor Symptoms in Parkinson's Disease: Implementation of a Portable, Computerized Measurement Tool

**DOI:** 10.4061/2010/760196

**Published:** 2010-06-30

**Authors:** Spyridon Papapetropoulos, Heather L. Katzen, Blake K. Scanlon, Alexandra Guevara, Carlos Singer, Bonnie E. Levin

**Affiliations:** ^1^Department of Neurology, University of Miami Miller School of Medicine, Miami, FL 33136, USA; ^2^Sierra-Pacific Mental Illness Research, Education, and Clinical Center, VA Palo Alto Health Care System, Palo Alto, CA 94304, USA; ^3^Department of Psychiatry and Behavioral Sciences, Stanford University School of Medicine, Stanford, CA 94305, USA; ^4^Department of Psychology, University of Miami, Coral Gables, FL 33124, USA

## Abstract

Quantification of neuromotor symptoms with device-based measures provides a useful supplement to clinical evaluation. Research using the CATSYS has established its utility as a computerized measurement system to quantify neuromotor function. The primary objective of this study is to provide technical guidance on the use of the CATSYS in Parkinson's disease (PD). Forty-four patients with idiopathic PD and 28 healthy controls were prospectively recruited and evaluated with CATSYS, a portable, Windows-based system consisting of a data logger and four different sensors (tremor pen, touch recording plate, reaction time handle, and force plate for balance recording) for quantification of neuromotor functions. CATSYS discriminated between PD and controls on measurements of rest/postural tremor, pronation/supination, finger tapping, simple reaction time, and postural sway intensity and velocity. CATSYS measurements using the proposed test battery were associated with relevant clinician-rated Unified Parkinson's disease rating scale (UPDRS) items assessing tremor and bradykinesia. More work is warranted to establish CATSYS as a diagnostic/monitoring instrument in movement disorders using the proposed technical approaches.

## 1. Background

Quantification of neuromotor symptoms such as tremor, bradykinesia, and imbalance using objective, device-based measures provides a useful supplement to clinical evaluation. Research using the Coordination Ability Test System (CATSYS, Danish Product Development Ltd., Denmark) has established its utility as a sensitive computerized measurement system to quantify normal and abnormal (clinical and subclinical) neuromotor function. Normative data has been obtained on 150 healthy men and women across five age groups, all of whom were shown to be free of neurologic deficits at the time of testing [1]. CATSYS has been used to assess the subtle neurological impact of exposure to toxic agents [2, 3]. It has also been shown to detect differences in dominant and nondominant hand tremor intensity among tobacco users and nonusers in a cohort of male industrial workers [4]. More recently, CATSYS has been used in studies of neurologic and neurodegenerative disorders with increasing frequency. Preliminary studies in patients with Parkinson's disease (PD) have shown the ability of CATSYS to discriminate pathologic from physiologic (normal) tremor in PD patients versus healthy controls [5]. Using bilateral measurements taken with CATSYS, Farkas et al. [6] were able to distinguish between the two most common tremor types, parkinsonian and essential tremor (ET), even at low tremor intensities. Our group has published the results of a pilot study of pre- and intraoperative objective quantification of parkinsonian symptoms during high-frequency deep brain stimulation (DBS) using permanently implanted brain electrodes in the subthalamic nucleus for the treatment of PD [7]. With the present work, we aim to provide our readers with technical guidance for measuring parkinsonian symptoms with CATSYS. We also present comparisons and reference measurements for PD patients and controls.

## 2. Methods

### 2.1. Sample

We evaluated 44 patients with idiopathic Parkinson's disease (PD) recruited from the Movement Disorders Clinic at the University of Miami Miller School of Medicine. A diagnosis of PD was made according to the UK PD Society Brain Bank criteria [8]. We also recruited a group of 28 consecutive healthy volunteers for a comparison group. Control participants were not matched to PD participants, due to the consecutive nature of recruitment in the departmental clinic. However, specific efforts were made to recruit healthy controls over 50 years old. All participants were administered the CATSYS neuromotor battery (participants with PD and motor fluctuation were evaluated in the “ON” state) and underwent comprehensive neurologic evaluation. Clinical assessment of PD participants also included the Unified Parkinson's Disease Rating Scale (UPDRS) III [9] (motor examination) during the “ON” state. PD patients were on stable antiparkinsonian treatment and received regular care from neurologists specializing in movement disorders. The local institutional review board approved the study, and all the participants gave written informed consent. Demographic and clinical characteristics are presented in Table 1.

### 2.2. Device

**Figure 1:**
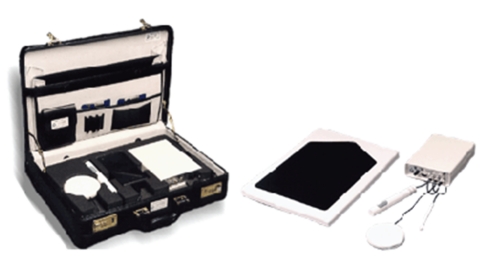
The Coordination Ability Test System (CATSYS) including a data logger and four different sensors: a tremor pen, a touch recording plate, a reaction time handle, and a force plate for balance (static posturography) recording.

The Coordination Ability Test System (CATSYS 2000; Danish Product Development, Ltd., Snekkersten, Denmark, http://www.catsys.dk) is a portable, Microsoft Windows-based system that consists of a data logger and four different sensors: a tremor pen, a touch recording plate, a reaction time handle, and a force plate for balance (static posturography) recording. The system quantifies numerous neuromotor functions including tremor (rest and postural/action), reaction time, bradykinesia, and postural sway. All collected data is easily transferred to a PC, displayed visually for the examiner, and readily exported for statistical analysis. The system is highly portable and fits in a briefcase with a total weight of less than 10 kg (see Figure 1). The entire CATSYS evaluation can be carried out in 30 minutes.

### 2.3. Tremor Pen

The Tremor Pen stylus (12 cm × 0.8 cm) contains a biaxial microaccelerometer and is sensitive in a plane perpendicular to the pen axis. Through the pen's connection to the datalogger, vibrations in the patient's extremities are recorded and displayed in real-time. Fast Fourier transformation analysis determines the normalized power distribution of the tremor in the frequency band 0.9 to 15.0 Hz. Quantifications generated from acceleration data are based on the Fourier power. Four key variables are calculated using the CATSYS software: (a) Tremor intensity, (b) Center frequency, (c) Frequency dispersion, and (d) Harmonic index. Tremor Intensity (TI) is the root mean square of acceleration recorded in the 0.9 to 15 Hz band. TI is measured in m/s^2^; larger values are indicative of more intense tremor. Center frequency (F50) is the median frequency of the acceleration in the 0.9 to 15 Hz band. A typical PD resting tremor is between 4 and 6 Hz [10]. Frequency dispersion (SF50), or the standard deviation (SD) of center frequency, represents the degree of irregularity of the tremor. A regular tremor has little frequency dispersion, indicating that most of the oscillations are within a narrow frequency band. Harmonic index (HI) compares the tremor frequency pattern with that of a single harmonic oscillation; a single harmonic oscillation has an HI of 1.0. HI decreases when the tremor is composed of frequent, irregular oscillations. For typical, homogenous parkinsonian resting tremor, the HI is close to 1.0. The tremor pen can be used to measure both resting and postural/action tremor.

### 2.4. Touch Recording Plate

The circular Touch Recording Plate (10 cm in diameter) is a round drum which connects to the datalogger and records each contact with the plate via a sensitive microphone. Using the CATSYS software, the (a) Mean frequency (Hz) of touches (MFT) and the (b) SD of the mean frequency (MFT-SD) are calculated for each task. The MFT is calculated as the total number of hits divided by the recording time and is measured in Hz. The MFT-SD is the deviation of hits from the average frequency calculated metronome hits. A person who is able to maintain the same hit speed will have a small deviation from the average frequency as opposed to a person with a variation in the hit speed. The Touch Recording Plate can be used to measure bradykinesia with a pronation/supination and finger tapping task, as will be described below.

### 2.5. Reaction Time Handle

The Reaction Time Handle (17 cm long and 2.4 cm in diameter) connects to the datalogger which records the press/release of a black button. A mechanical click response is also provided as feedback for a successful response to the subject with each click. Audible signals are emitted from the speaker on the datalogger at random intervals during a fixed time period. Responses, which are simple clicks with the thumb, are recorded in respect to the time lapse between an audible signal and handle click. Reaction times less than 0.10 seconds are considered invalid and are discounted by the software. Reaction times larger than a user-specified “maximum reaction time” are also excluded. The maximum permitted reaction time is a predefined waiting period. After each stimulus, the system waits this amount of time for the reaction time handle to be clicked. If no click occurs, the next period at which a stimulus is randomly emitted begins. Using the CATSYS software, the (a) Mean reaction time (RT; measured in seconds) and the (b) SD of the reaction time (RT-SD) are calculated for the task. This device can be used to measure simple reaction time, as will be described below.

### 2.6. Force Plate

The force plate is a rectangular static posturography plate (40 cm in length and 29 cm in width) that connects to the datalogger and records changes in vertical force. The plate records weight measured in kilograms. Forces along the vertical plane are recorded in three points to determine the position of the center of force on the plate. The movement of the force center in the XY-plane defined by the surface of the force plate is recorded in time and analyzed to provide key variables. Transversal Sway (TS) and Sagittal Sway (SS) are measured in mm and are defined as the mean of the *x*- and *y*-axis values of the force center in a coordinate system with the mean force center position as *x* = 0, *y* = 0. The Mean Sway (MS), defined as the simple mean of the distance from the geometrical mean for center position to all recorded force positions, is also measured in mm. The Sway Area (SA), defined as the area of the smallest polygon created by changes in force along the *x*- and *y*-axes, includes the total trajectory of the force center in the horizontal force plate plane. The SA is calculated by looking at the force center trajectory through a grid. This unit is measured in mm². Sway Velocity (SV), the average travel speed of the force center in the horizontal force plate plane, is calculated by dividing the total length of the force center trajectory (mm) by the recording period length (s). This unit is measured in mm/s. Sway Intensity (SI) is calculated by taking the root mean square of acceleration recorded in the 0.1 Hz to 10.1 Hz band. SI is measured in mm/s^2^. The force plate can be utilized to measure postural stability, as will be defined in detail below.

## 3. CATSYS Evaluation Protocol

### 3.1. Resting Tremor

With the hand in a relaxed position, the tremor pen was inserted between the index and middle finger bordered by the thumb on the opposite side. Participants were instructed to count backwards as a distracter task. Resting tremor was measured for 8.2 seconds in each hand, successively.

### 3.2. Postural Tremor

The tremor pen was inserted between the index and middle finger while lying on the thumb. Participants were asked to hold the pen horizontally, at eye level, approximately 10 cm from the nose with their elbow joint at a right angle and free from the body. Participants were instructed to count backwards as a distracter task. Postural tremor was measured for 8.2 seconds in each hand, successively.

### 3.3. Pronation/Supination

The touch recording plate was placed on a flat surface next to the patient. Participants were asked to tap, with their hand in an alternating pronation/supination pattern, on the plate, as fast as possible with the recording plate on a table in front of them. Performance on the maximum frequency pronation/supination task was measured for 10 seconds for both the dominant and non-dominant hand.

### 3.4. Finger Tapping

The touch recording plate was placed on a flat surface next to the patient. While seated with the recording plate on a table in front of them, participants were asked to position their thumb and middle finger to straddle the touch recording plate with the wrist resting on the table. Participants were asked to tap on the plate with their index finger as quickly as possible, while raising the index finger as far off the plate as possible between each tap. Performance on the maximum frequency finger tapping task was measured for 10 seconds for the dominant and non-dominant hand.

### 3.5. Simple Reaction Time

The reaction time handle was placed in the patient's hand, with the thumb situated over the black “click” area. Participants were instructed to respond to a series of randomly sequenced audible tones, as fast as possible, by clicking the black area on the handle. Performance on simple reaction time was measured in response to nine randomly spaced tones during a 40-second time period for both the dominant and non-dominant hand.

### 3.6. Postural Stability

The Force Plate was placed on a flat, hard surface, and the participants were asked to stand on the plate for a period of 80.5 seconds with their eyes open. Recording began after a 10- second run-in period. There was also a five-second run-out period, so that the recording time for PI was 65.5 seconds. This task was only completed in the outpatient setting.

## 4. Statistical Analysis

Spearman correlation coefficients were used to examine the relationship between UPDRS-III motor scores and CATSYS measurements. Mann-Whitney *U* tests were used for group comparisons between PD and healthy control groups, as CATSYS measurements for the PD group violated normality assumptions. The most affected side (i.e., the side with the worst task performance) of the PD participants (identified for each task) was compared with the performance of the worst performing side of the healthy control group for all upper extremity measures. A probability value (*p*) of ≤.05 was considered significant for all analyses. Statistical analyses were performed using SPSS for Windows 17.0 (SPSS Inc., Chicago, IL).

## 5. Results

### 5.1. Relationship of CATSYS and UPDRS

CATSYS values were collected for upper extremity tremor (resting and postural), bradykinesia (pronation/supination and finger tapping), and reaction time. Postural sway data was collected for a subset of PD participants (*n* = 7) and healthy controls (*n* = 10).

### 5.2. Resting Tremor

Both right and left CATSYS resting TI scores were significantly correlated with the right and left values for UPDRS item 20 (Right *ρ* = .680, *p* < .001; Left *ρ* = .739, *p* < .001).

### 5.3. Postural Tremor

Both right and left CATSYS postural TI scores were significantly correlated with the right and left values for UPDRS item 21 (Right *ρ* = .803, *p* < .001; Left *ρ* = .793, *p* < .001).

### 5.4. Bradykinesia

The mean value for CATSYS pronation/supination ([R + L]/2) was calculated and was significantly correlated with UPDRS item 31 (*ρ* = −.411, *p* < .014). CATSYS finger tapping values for the left and right hand show no relationship with UPDRS bradykinesia items (all *p* *s* > .05).

### 5.5. CATSYS Discriminates between PD and Control Subjects

The PD group demonstrated greater resting TI (*U* = 278.0, *p* < .001) and a higher HI (*U* = 379.5, *p* < .007) than healthy controls. Postural TI (*U* = 164.5, *p* < .001) was also greater in PD. The PD group exhibited slower pronation/supination movements (*U* = 125.5, *p* < .001) and a lower SD of pronation/supination frequency (*U* = 129.5, *p* < .001) than healthy controls. Finger tapping (*U* = 178.0, *p* < .005) and reaction time (*U* = 275.0, *p* < .009) were slower in PD compared to controls. In the subset of participants who underwent the static posturography assessment, the PD group showed more sway in the sagittal plane (*U* = 13.5, *p* < .037) and demonstrated greater overall sway intensity (*U* = 9.0, *p* < .012) and velocity (*U* = 8.0, *p* < .009) than healthy controls. All other standard CATSYS measurements failed to discriminate between PD and healthy controls. Means and standard deviations for all CATSYS values are presented in Table 2.

## 6. Discussion

This study provides technical guidance on the use of the CATSYS and proposes a battery of tests to help investigators obtain uniform/standardized data. The CATSYS system demonstrated clinically relevant measurement feasibility of selected parkinsonian symptoms relative to the UPDRS. These preliminary data suggest that the CATSYS system is able to discriminate between PD patients and healthy individuals without evidence of disordered movements. The purpose of the current study was not to evaluate the diagnostic utility of the CATSYS system, but to describe how it can be used to provide objective measurements of parkinsonian symptoms. CATSYS offers an opportunity to quantify objectively several parkinsonian motor symptoms and supplement clinician observation. Unlike traditional clinical rating scales that utilize categorical ratings, the CATSYS quantifies motor performance on a continuum, allowing for greater precision in recording subtle change in PD motor symptomatology.

### 6.1. CATSYS Versus Other Computerized Assessment Tools

Other methods are available which permit the objective assessment of motor symptomatology. There are at least eight systems that have been published with data demonstrating the ability to quantify PD motor symptoms [7, 11–17]. Each system utilizes a different methodology and a unique set of strengths and weaknesses. Compared to other available tools, CATSYS offers the ability to assess a wider range of symptoms and to date is the only system available that has been used to measure lower extremity symptoms [18]. The CATSYS also offers the ability to quantify postural instability, a key feature of advanced PD not measured by the other available systems. Other major advantages of the CATSYS include the availability of normative data, portability, and ease of administration.

## 7. Conclusions

Using a noninvasive, simple, and sensitive electronic recording method of symptom registration, we were able to objectively quantify parkinsonian neuromotor symptoms and provide reference values that differentiate patients from control subjects. More work is warranted to establish CATSYS as a diagnostic or monitoring instrument in movement disorders using the proposed technical approaches

## Figures and Tables

**Table 1:** Clinical Demographic Data for Sample.

	PD	Control
	(*n* = 44)	(*n* = 28)
	*M (SD) *	*M (SD) *
Age	62.7 (9.5)	51.1 (16.9)
Gender: % M/F	71/29	54/46
Ethnicity: % Nonhispanic/Hispanic	51/49	57/43
Age of Onset	52.8 (11.3)	
Disease Duration	9.0 (5.4)	
Hoehn & Yahr Stage	2.3 (0.7)	
UPDRS-III Motor Score	21.7 (9.8)	

**Table 2:** CATSYS Values for PD and Control Participants.

	PD			Control		
	*N *	*M *	*SD *	*N *	*M *	*SD *

CATSYS Rest TI*	44	1.34	3.02	28	0.10	0.07
CATSYS Rest F50	44	6.63	2.33	28	6.84	3.08
CATSYS Rest SF50	44	2.22	1.34	28	2.61	1.16
CATSYS Rest HI**	44	0.92	0.06	28	0.88	0.07
CATSYS Postural TI*	44	1.75	3.20	18	0.21	0.30
CATSYS Postural F50	44	6.20	1.98	18	4.96	2.40
CATSYS Postural SF50	44	2.33	1.56	18	2.93	1.48
CATSYS Postural HI	44	0.91	0.06	18	0.89	0.06
CATSYS Pronation/Supination (events/s)*	40	2.45	0.81	18	3.43	0.60
CATSYS SD of Pronation/Supination (events/s)*	40	1.29	0.34	18	1.65	0.23
CATSYS Finger Tapping (events/s)**	38	3.83	1.01	18	4.63	0.82
CATSYS SD Finger Tapping (events/s)	38	1.57	0.26	18	1.68	0.17
CATSYS Reaction Time (s)**	42	0.41	0.21	22	0.29	0.06
CATSYS SD of Reaction Time (s)**	42	0.11	0.08	22	0.07	0.05
CATSYS Mean Sway (mm)	7	7.83	5.24	10	4.90	1.16
CATSYS Transversal Sway (mm)	7	3.29	2.55	10	2.84	1.15
CATSYS Sagittal Sway (mm)**	7	6.53	4.09	10	3.40	0.87
CATSYS Sway Area (mm^2^)	7	1375.57	2816.46	10	188.60	91.62
CATSYS Sway Intensity**	7	9.15	8.57	10	3.40	0.70
CATSYS Sway Velocity (mm/s)**	7	12.89	7.43	10	7.24	1.67

*p < .001

**p < .05
